# Spatial Patterns of Dengue Incidence in Nepal During Record Outbreaks in 2022 and 2023: Implications for Public Health Interventions

**DOI:** 10.4269/ajtmh.24-0747

**Published:** 2025-06-10

**Authors:** Simrik Bhandari, Jason K. Blackburn, Sadie J. Ryan

**Affiliations:** ^1^ Quantitative Disease Ecology and Conservation Lab, Department of Geography, University of Florida, Gainesville, Florida;; ^2^ Emerging Pathogens Institute, University of Florida, Gainesville, Florida;; ^3^ Spatial Ecology and Epidemiology Research Laboratory, Department of Geography, University of Florida, Gainesville, Florida

## Abstract

Dengue, which was first reported as a travel case in Nepal in 2004, was initially confined to the lower plains but has spread to higher elevations. Large outbreaks in 2022 and 2023 (54,784 and 51,243 cases, respectively), reached every district (*n* = 77). We calculated the district-wise incidence for 2022 and 2023 by digitizing case data from Nepal’s Ministry of Health and Population and 2021 census data from the National Statistics Office. The incidence and peak incidence months for each year were mapped, and spatial clusters (hotspots and cold spots) and outliers of incidence rates were identified using Local Moran’s I. In 2022, district-wise peak cases occurred from August to October. One hotspot (high–high cluster), with high values surrounded by high-value neighbors, including six districts around Kathmandu, and one cold spot (low–low cluster), with low values surrounded by low-value neighbors, comprising eight high-elevation districts in Nepal’s northwest region were identified. In 2023, cases peaked from March to November, indicating more distributed peaks that started earlier; hotspots shifted to the north-central and eastern regions, and a low–high outlier district in the central region was identified. Identifying the timing of peaks and spatial clusters of dengue incidence can inform targeted management, thereby improving effectiveness and cost-efficiency. A baseline examination of recent dengue incidence in Nepal, highlighting timing and spatial clustering in incidence, is provided in this study. The mountainous northwest cold spots align with expectations of fewer mosquitoes because of the geography and climate. However, the 2022 dengue incidence peaked across all 77 districts in 3 months, suggesting that ecological and climatic factors may no longer be effective barriers.

## INTRODUCTION

Dengue fever, a mosquito-borne viral infection, is a significant global public health problem that results in an estimated 390 million infections annually.^1^ Dengue virus (DENV) is an arbovirus in the family Flaviviridae that is transmitted by *Aedes* spp. mosquitoes, primarily *Aedes aegypti* (*Ae. aegypti*) and *Aedes albopictus.*^2^ Dengue virus infection can present with a range of symptoms. Infected individuals may be asymptomatic, experience mild fever, or suffer from severe forms of the disease, such as dengue hemorrhagic fever or dengue shock syndrome. Dengue hemorrhagic fever is characterized by high fever, severe headache, bleeding (such as nosebleeds or gum bleeding), and abdominal pain. Dengue shock syndrome, which is a more severe progression, includes symptoms like a rapid, weak pulse, cold, clammy skin, and low blood pressure, leading to potentially life-threatening shock. Dengue is also known as break-bone fever because of the severe muscle and bone pain it can cause.^3^ The disease is more prevalent in warmer regions near the equator, particularly in tropical and subtropical areas.^4^^,^^5^ Dengue virus has four distinct serotypes (DENV-1, DENV-2, DENV-3, and DENV-4). Infection with one serotype provides immunity to that specific serotype but increases the risk of severe disease if a person is subsequently infected with a different serotype.^6^ Furthermore, there is evidence that immunity to infecting serotypes is not lifelong.^7^ These phenomena complicate the development of an effective dengue vaccine.

Dengue impacts livelihoods, and the burden of the disease affects healthcare systems, especially in regions with tropical and subtropical climates.^4^^,^^5^ In Nepal, dengue fever was first reported in 2004 and has evolved into a significant public health issue. It is now considered endemic in this country. All four DENV serotypes are now present and cocirculating in Nepal, and both *Aedes albopictus* and *Ae. aegypti* have been identified as the key transmission vectors in this country.^8^

Initially confined to the warmer southern lowland Terai region of Nepal, dengue has spread to higher elevations and previously unaffected areas.^9^^,^^10^ Climate change has been shown to have influenced mosquito breeding patterns and expanded *Aedes* spp. mosquito habitats in Nepal.^11^^,^^12^ Additionally, excessive rainfall, poor waste management, and rapid unplanned urbanization create ideal breeding grounds for these mosquitoes. Urbanization supports mosquito breeding habitats by creating diverse artificial aquatic habitats, such as buckets and flowerpots, which provide ideal conditions for *Aedes *spp.^13^^,^^14^ These processes have been underway in Nepal, as in much of the world, over the past two decades. The open border with India also plays a role in dengue transmission in Nepal, facilitating cross-border introductions and the transmission of the disease.^15^

**Figure 1.**
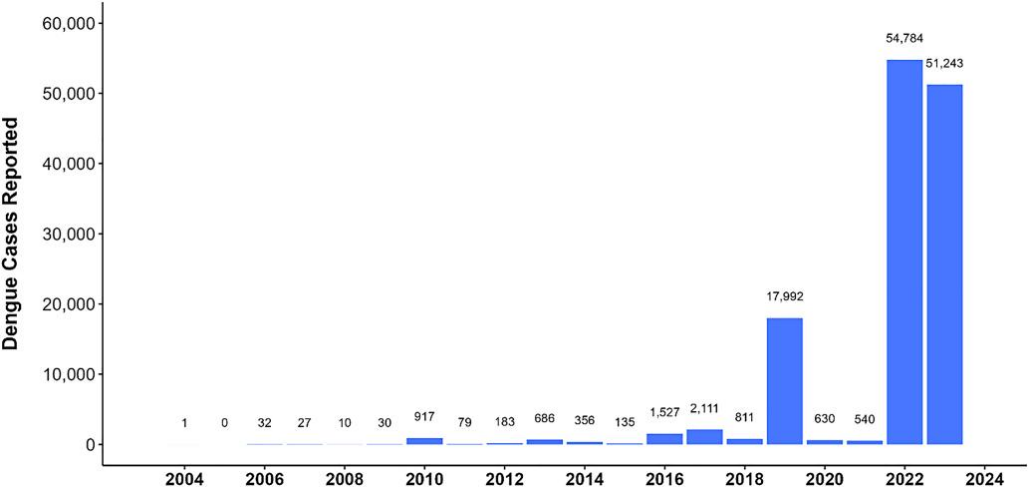
Annual reported dengue cases in Nepal.

Nepal’s health system faces considerable challenges in controlling dengue. These include difficulties in monitoring, early detection, and diagnosis. Significant outbreaks in 2010, 2013, 2016, 2019, 2022, and 2023 highlighted these challenges.^16^ In 2020 and 2021, there was a decrease in reported cases, likely due to lockdowns and restricted movement; however, underreporting due to a focus on coronavirus disease 2019 may have played a role.^17^ A significant rise in dengue in Nepal was observed in 2022, with an alarming 54,784 cases recorded.^18^ In 2023, although there was a decrease in the number of reported cases, the number was still high at 51,243 (Figure 1). The cocirculation of DENV-1, DENV-2, and DENV-3 occurred during both years.^19^^,^^20^ In response to an increasing number of cases and recent record years, the government has taken steps to mitigate outbreaks by offering free testing and treatment for dengue and focusing on prevention and control measures.^21^

Identifying which regions are experiencing higher or lower than expected disease incidence can inform targeted public health interventions like mosquito control programs and public health education, thereby ensuring efficient resource allocation in high-risk zones. Geospatial methods are crucial to studying disease incidence and spread, particularly in a management context.^22^^,^^23^ The local indicators of spatial association (LISA) statistic is often used in exploratory spatial data analysis (ESDA) frameworks to quantify spatial clustering or the dispersion of disease cases or incidence in a study area, including studies on dengue and other arboviral diseases in emerging and endemic settings.^24^^,^^25^ Identifying spatially discrete areas of significantly high (e.g., hotspots) or low (e.g., cold spots) disease activity within administrative functional boundaries can frame responses to public health events. In this study, we map the district-level dengue incidence in Nepal in 2022 and 2023 and identify peak case months by district, as well as patterns of spatial heterogeneity in incidence across the country, using LISA methods.

## MATERIALS AND METHODS

### Study area.

**Figure 2.**
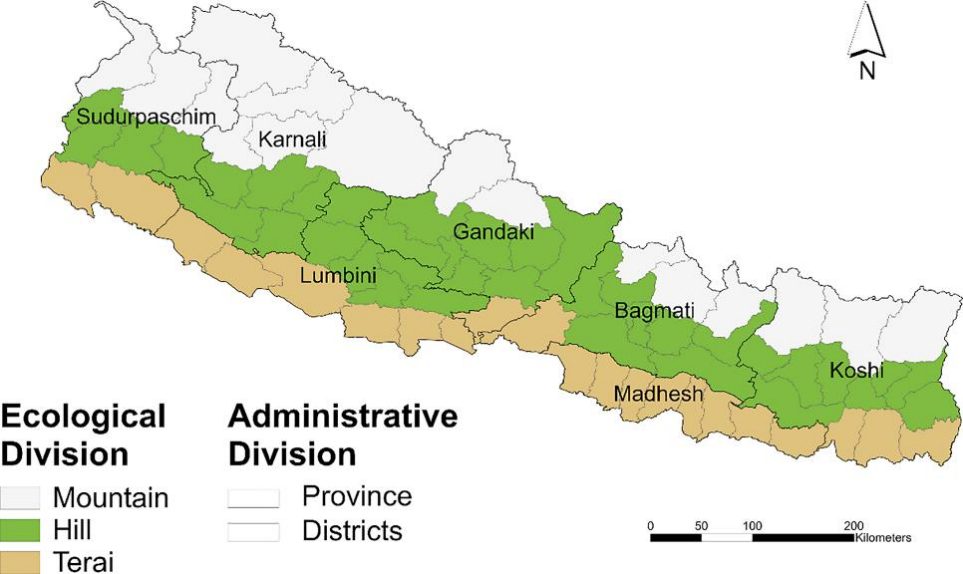
Ecological and administrative divisions of Nepal. The 77 districts and seven provinces of Nepal, overlaid on three ecological divisions: Mountain (white), Hill (green), and Terai (brown).

Nepal is a small (147,516 km^2^), mountainous, landlocked country in South Asia that is surrounded by China to the north and India to the south, east, and west. The population in 2021 was 29,164,578, with a population growth rate of 0.92%.^26^ The country exhibits diverse topography and ecology that can be broadly divided into three geographic regions: Terai, Hill, and Mountains. Each region exhibits distinct climatic conditions, as categorized by the Koppen–Geiger climate classification (Figure 2).^27^^,^^28^ The low-lying Terai plains in the south, with an elevation of 60 to 330 m above sea level (asl), experience a tropical savannah climate characterized by hot summers, mild winters, and monsoonal rainfall, receiving ∼80% of their annual precipitation during the summer monsoon. Moving northward, the hilly midlands, with an elevation of 330 to 3,500 m asl, experience a temperate climate with two main subtypes: temperate with dry winters and hot summers and temperate with dry winters and warm summers. The high Himalayan mountains, with an elevation greater than 3,500 m asl in the north, experience cold and polar climates in which temperatures drop significantly, and winters are harsh, with prolonged snow cover, according to the Koppen–Geiger climate classification.^28^ Nepal experiences four distinct climatic periods during the year: the pre-monsoon season (March to May), the monsoon season (June to September), the post-monsoon period (October to November), and the winter months (December to February).^29^ Administratively, Nepal is currently divided into seven provinces and 77 districts.

### Data acquisition and processing.

Monthly district-level dengue cases reported in 2022 and 2023 were obtained from the Epidemiology and Disease Control Division of the Ministry of Health and Population, Nepal.^30^^,^^31^ Population data for each district were obtained from the National Census Report conducted in 2011^32^ and 2021.^26^ Administrative division shapefiles (geographic coordinate system WGS 1984) were downloaded from the National Geoportal (Survey Department, Government of Nepal, Kathmandu; https://nationalgeoportal.gov.np/). Dengue cases and population datasets were digitized and geospatially joined to district polygons for analysis and visualization using ArcGIS Pro (Esri, Redlands, CA).^33^

#### Population projections.

District-level population estimates for 2022 and 2023 were projected by using 2011 and 2021 census data and an exponential growth model.^34^^,^^35^ The annualized growth rate (*r*) was calculated as follows:

$$r=\text{ln}\left(P_{2}/P_{1}\right)/t,$$

where *P*_1_ is the 2011 population count, *P*_2_ is the 2021 population count, and *t* is the number of years between the two censuses.

This growth rate was then used to project the population for the required years as follows:

$$P_{x}=P_{2}.e^{rt},$$

where *P_x_* is the population estimate for the target year (*x*), and *t* is the number of years between the 2021 census and the target year (2022, 2023).

#### Case timing peaks.

The months of peak case numbers, by district, were recorded for 2022 and 2023 and geospatially joined to district polygons for mapping.

#### Incidence rates.

The annual dengue incidence rates per 100,000 people were calculated using GeoDa 1.20 (Center for Spatial Data Science, University of Chicago, IL).^36^ Dengue incidence was calculated as the total number of dengue cases in a reporting year in each district divided by its total population.

$$\text{Incidence}\;=\frac{\text{Dengue}\;\text{cases}\;\text{in}\;\text{the}\;\text{district}}{\text{Total}\;\text{population}\;\text{in}\;\text{the}\;\text{district}}\times 100,000$$

#### Spatial clustering.

Global Moran’s I provides an indicator of spatial clustering to assess whether a phenomenon is spatially random, clustered, or dispersed on a landscape. This index takes values of –1 to 1, from dispersed to clustered. Through simulations, permutations can provide a distribution of values, such that a statistic of significance can be assessed. This method was applied to the district-wise incidences in 2022 and 2023, and 999 permutations were run in GeoDa software to derive z-scores and assess significance at 0.025, accounting for the 2 years in this analysis.

#### LISA analysis.

The local Moran’s I statistic is a popular tool that is used in spatial analysis to identify disease patterns.^37^^,^^38^ It is used to determine if similar or dissimilar values in a dataset are geographically clustered to detect the locations of of hotspots, cold spots, and outliers.^39^ Hotspots are clusters that contain areas with high values surrounded by high-value neighbors (high–high), indicating regions with increased disease incidence. Cold spots are clusters that contain areas with low values surrounded by low-value neighbors (low–low), representing areas with consistently low disease incidence. Spatial outliers occur when a high value is surrounded by low values (high–low) or a low value is surrounded by high values (low–high), suggesting areas with patterns that differ from their surroundings. When working with small population areas, rate data, such as incidence, can be subject to instability because of small populations in subregions. Smoothing techniques in spatial analysis are applied to adjust rates to account for this instability.^40^ To assess any potential rate instability in this study, both empirical Bayes smoothing and spatial Bayes smoothing were applied to the incidence rates and compared with raw rates by using box plots (Supplemental Figure 1).

Local Moran’s I was calculated for the raw incidence rate by using a queen contiguity spatial weight matrix with 999 permutations at a 0.025 significance level, which was adjusted using the Bonferroni correction to account for analyses spanning 2 years, thereby identifying significant clusters and outliers. We used the queen contiguity matrix because our data were aggregated to the district level, and our focus was on neighboring districts. Maps of significant hotspots and cold spots for each year were constructed to describe spatial patterns for each year.

## RESULTS

**Figure 3.**
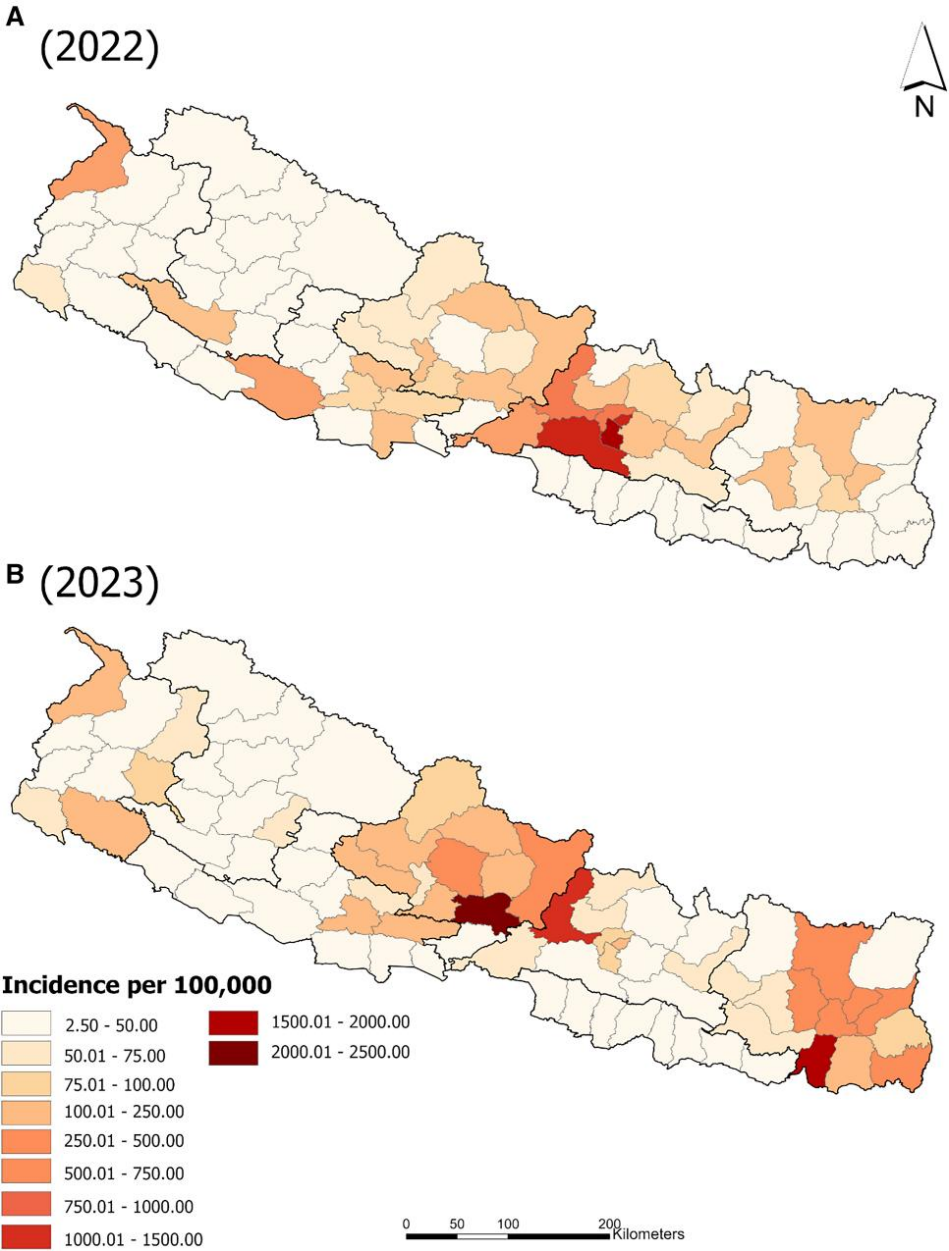
Incidence per 100,000 people by district of Nepal in 2022 and 2023.

In 2022, in Nepal, there was a total of 54,784 reported dengue cases, and the district-level incidence ranged from a low of 4.21 to a high of 1,716.89. In 2023, the total number of reported cases was 51,243, with a district-level incidence range of 2.49 to 2,242.44. The districts with the highest incidence rates were not identical between the two years, and in 2023, high incidence rates occurred further east than they did in 2022 (Figure 3).

**Figure 4.**
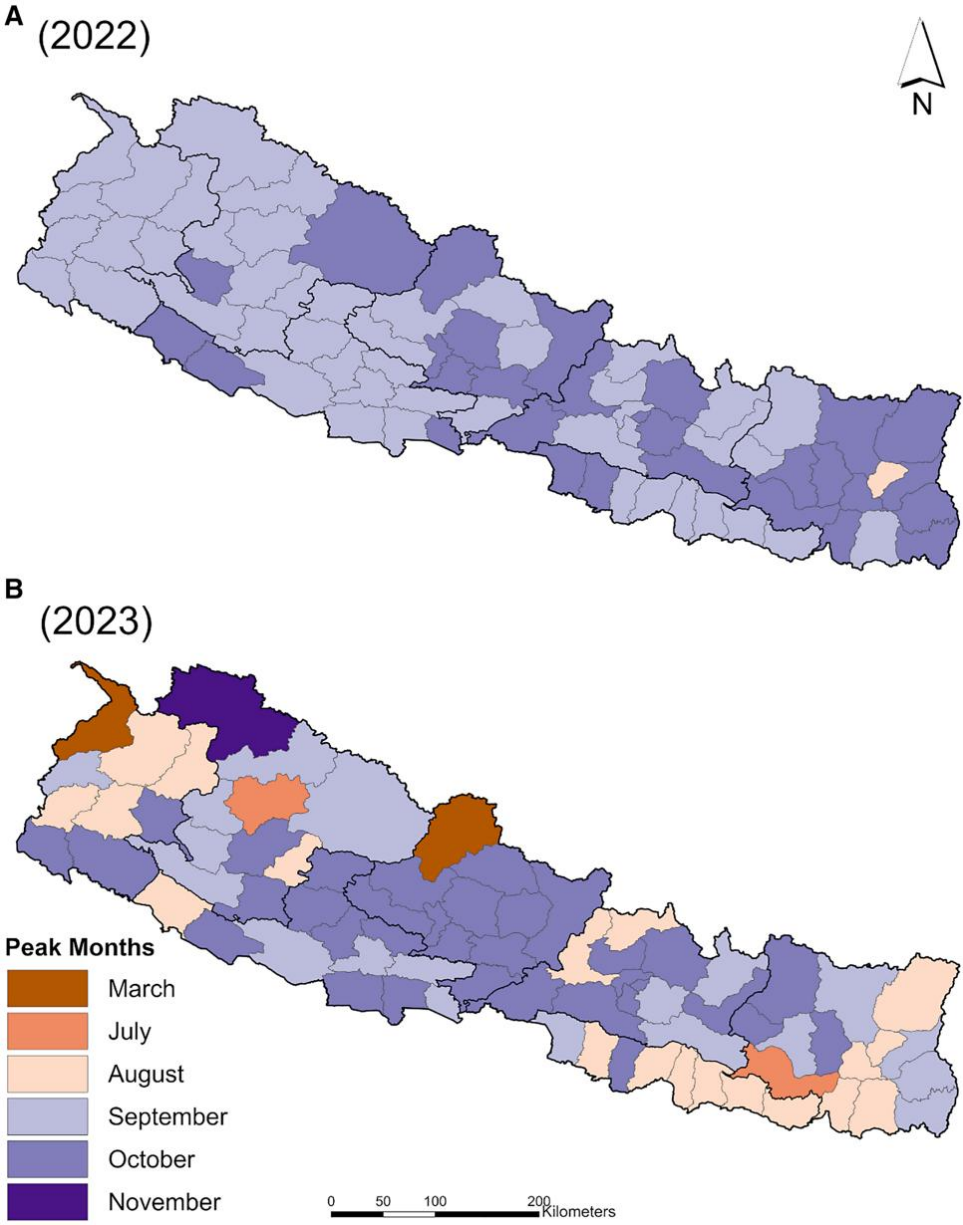
Months with peak numbers of dengue cases by district in 2022 and 2023.

The district-level peak case month map for 2022 illustrates that most dengue cases were reported in the months of August, September, and October (Figure 4A), with no obvious spatial patterns, suggesting that the whole country experienced one epidemic peak season. In 2023, case numbers were at their highest in some districts as early as March and peaked as late as November in the mountainous region (Figure 4B). The pattern of peak months in 2023 was more heterogeneous, although this was only visually assessed.

Global Moran’s *I* indicated clustering in both 2022, but not in 2023, with positive values of *I* (Table 1). Based on permutation tests, data from 2022 revealed significant clustering at the global level for our criterion of significance level (α = 0.025), whereas the 2023 value (*I = *0.095) was not significant.

**Table 1:** Global Moran’s* I* values for dengue incidence, aggregated by district for 2022 and 2023

Year	Total Dengue Cases	Moran’s I*	*Z*-Score	*P*-Value
2022	54,784	0.472	7.4798	**0.001**
2023	51,243	0.095	1.7947	0.060

*P*-values in bold indicate statistically significant results.

* Moran’s *I* values range between –1 and 1, where negative values indicate dispersion and positive values indicate clustering.

**Figure 5.**
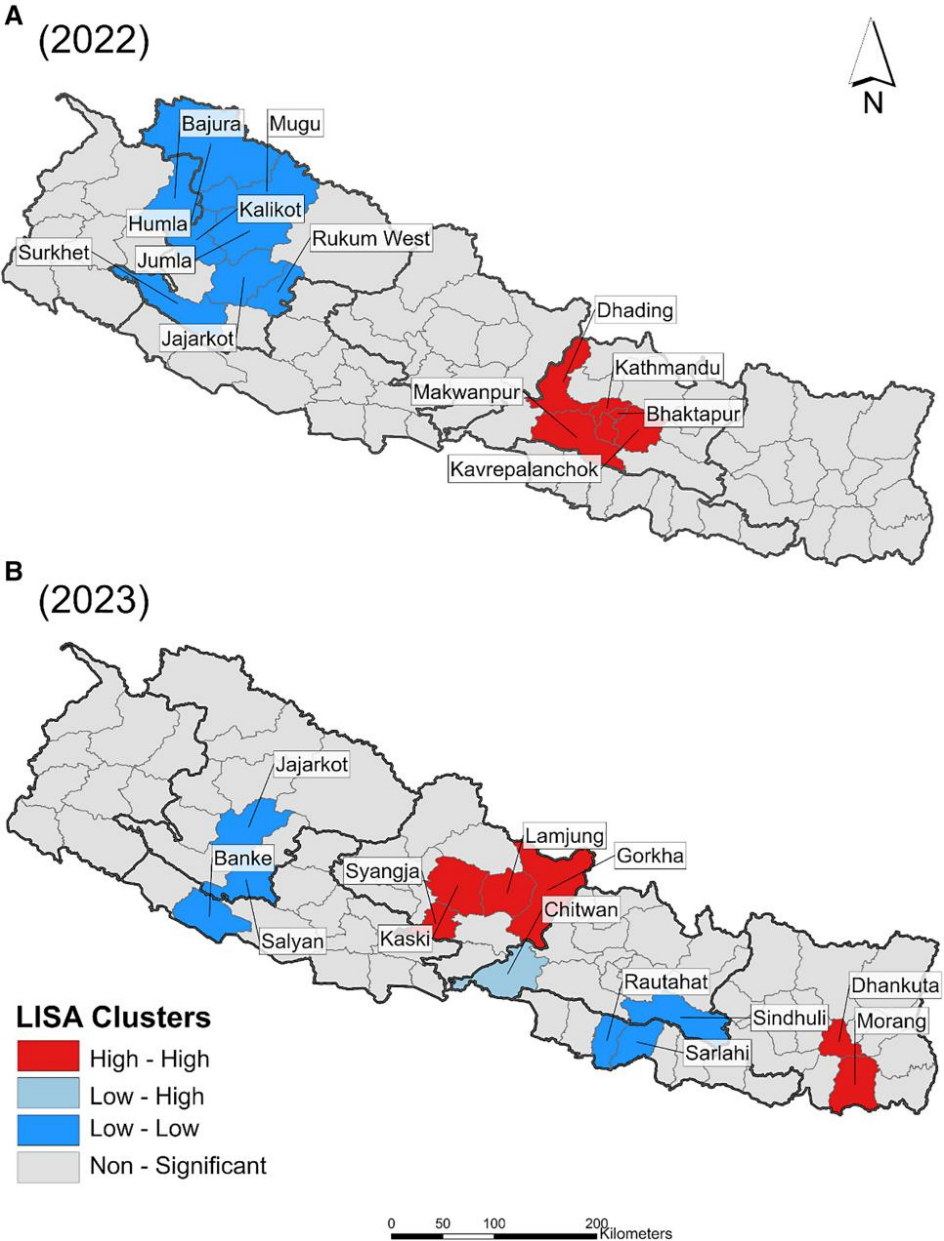
High- and low-incidence areas of dengue for 2022 and 2023 in Nepal, as identified using the local indicator of spatial association. Red areas indicate clusters of districts in which districts with relatively higher rates are surrounded by districts with similar relatively higher rates, indicating a significant hotspot for dengue transmission. Blue areas represent low clusters, indicating districts with relatively lower rates surrounded by districts with similar lower rates. The light blue area indicates a statistically significantly lower incidence than expected in the neighboring districts.

The LISA map for the 2022 dengue incidence (Figure 5A) reveals one hotspot and one regional cold spot. Six districts in the centrally located province of Bagmati (Kathmandu, Lalitpur, Bhaktapur, Kavrepalanchowk, Makwanpur, and Dhading) exhibited statistically higher dengue incidences, and eight districts exhibited statistically lower dengue incidences in the west (Humla, Mugu, Bajura, Kalikot, Jumla, Jajarkot, Rukum West, and Surkhet).

The LISA map for the 2023 dengue incidence reveals a shift in clusters (Figure 5B) to two hotspots and two cold spots. Four centrally located districts (Gorkha, Lamjung, Kaski, and Syangjha) and two districts located to the east (Dhankuta and Morang) exhibited statistically higher dengue incidences. Similarly, three districts in the west (Jajarkot, Salyan, and Banke) and three centrally located districts (Sindhuli, Sarlahi, and Rautahat) exhibited statistically lower dengue incidences. One low–high outlier in the Chitwan District also exhibited a lower incidence than that of neighboring districts.

## DISCUSSION

In 2022 and 2023, Nepal experienced significant dengue outbreaks, reporting more than double the number of cases of any previous year on record. In a country experiencing such rapid and expansive outbreaks of this emergent vector-borne disease, visualizing and understanding spatial patterns and timing can inform public health planning and intervention. Dengue cases were reported in every district in the country in 2022, which indicates that previously assumed climate and ecological barriers to establishment and transmission are no longer curbing its spread. By using exploratory geospatial approaches, we digitized and visualized the 2022 and 2023 dengue outbreak years for Nepal on the basis of published case data and census population projections.

By mapping the peak case months by district, we showed that in 2022, the highest case numbers occurred clustered in time, spanning from August to October, with no obvious heterogeneity across the country. However, in 2023, peak case numbers occurred in the districts from March to November, with the mountainous districts peaking both earliest and latest and exhibiting a patchier distribution of peak months across the country. Annual incidence maps revealed that in 2022, very high-incidence districts were largely clustered around Kathmandu. In 2023, however, not only were these not the same districts exhibiting higher incidence, but there was also an emergence of higher reported incidence in eastern Nepal. The distribution of incidence was clustered globally in 2022, but not in 2023. This finding was echoed in a shift in dengue hotspots and cold spots from 2022 to 2023. In 2022, the hotspot (high–high cluster of districts) identified around Kathmandu was also contained entirely within the Bagmati Province, whereas in 2023, the two identified hotspots were clusters of districts occurring in the two provinces to the west (Gandaki) and east (Koshi). Interestingly, one district in Bagmati became part of a cold spot in 2023, suggesting lower incidence than expected, and a second district in Bagmati (Chitwan) was a low–high outlier in 2023, indicating incidence rates that were significantly lower than expected and not part of a cluster. Although we only report clusters that meet our Bonferroni corrected significance criterion for the two years examined together here (*P* = 0.025), we include a supplemental figure (Supplemental Figure 2) that shows the LISA results at significance levels of *P* = 0.05*, P* = 0.01*, *and *P* = 0.001. Determining if province-level shifts, such as those reported here, could reflect the impact of population-level immunity from the previous year at the district level or successful interventions requires follow-up studies. Improved vector control and prevention necessitate approaches outside the scope of this study, including serological surveillance and cross-sectional or longitudinal health center-level survey instruments.

Nepal’s climate has been changing in recent decades, with rising temperatures, more frequent heat extremes, and shifts in precipitation patterns, which may have affected the sizes and patterns of dengue outbreaks. In 2022 and 2023, the average mean surface air temperatures in Nepal remained high (13.25°C in 2022 and 13.17°C in 2023), and the number of very hot days (heat index >35°C) increased from 12.99 to 13.93. In these two years, changes in the precipitation pattern were also reported. In 2022, Nepal experienced 97.3% of normal monsoon rainfall, but the rainfall dropped below normal levels in mid-July. In 2023, the total precipitation was lower, at 91.2% of normal, making it the eighth driest year since 1981. However, some months experienced more rain than usual, including June 2022, March 2023, and October 2023, which may have influenced mosquito breeding seasons.^41^^–^^43^

It is important to note that this study relies on reported case data, and in a large and rapidly unfolding outbreak situation, the availability of diagnostic testing, accessibility to health facilities and implementation of interventions are unevenly distributed in space and time, which may lead to uneven data reporting and the over-representation of areas with more resources. Although we might expect that areas with very sparse populations, where healthcare access is quite limited, such as the mountainous region, could lead to a lack of reporting, the presence of cases reported in every district in 2022 suggests that this lack did not lead to overly spatially biased outcomes in our study. However, we are cautious in our interpretation of the cold spot cluster of low–low incidence in the northwest mountainous region identified in 2022 because it may be more indicative of sparse reporting than existing prevention. Although the sparse population in the mountainous districts could also distort incidence rate calculation, we found that the incidence rates were not unstable or impacted by population distribution for the purposes of conducting the ESDA analyses in this study.

By using LISA methods, we identified hotspots (i.e., districts with higher dengue incidence), indicating potential high-priority areas for targeted health measures in those regions. In the scenario of vector-borne disease management, these hotspots can indicate areas that may need increased intervention, such as spraying, larval source management, or the increased allocation of hospital resources for palliative care and diagnostic testing. The shifts in identified hotspots between the two years in this study are worth further consideration to explore how best to allocate limited resources. We also highlighted cold spots, which are regions with lower-than-expected dengue incidence that could result from variations in local health strategies, population immunity, or case reporting.

Nepal is a small country with a diverse topography, which leads to a sharp climate and ecological gradients. It is experiencing rapid shifts in climate-induced impacts, which influences both human population distributions on the landscape and the ecology of vector habitats. Understanding the dynamics that underpin the spatial patterns of a rapidly emerging vector-borne disease, as described here through the analysis of spatial human incidence data, requires further investigation. Future research on the spatial distribution of dengue in Nepal should explore additional drivers (socioeconomic, climatic, and environmental influences) of dengue transmission.

## Supplemental Materials

10.4269/ajtmh.24-0747 Supplemental Materials
