# Supplementary material for: Spatial Patterns of Dengue Incidence in Nepal During Record Outbreaks in 2022 and 2023: Implications for Public Health Interventions
Source: Am J Trop Med Hyg. 2025 Jun 10;113(2):366–73. doi: 10.4269/ajtmh.24-0747 (PMC12360065; doi:10.4269/ajtmh.24-0747)
Supplement: Supplemental Materials [file tpmd240747.SD1.pdf]

Supplementary Material

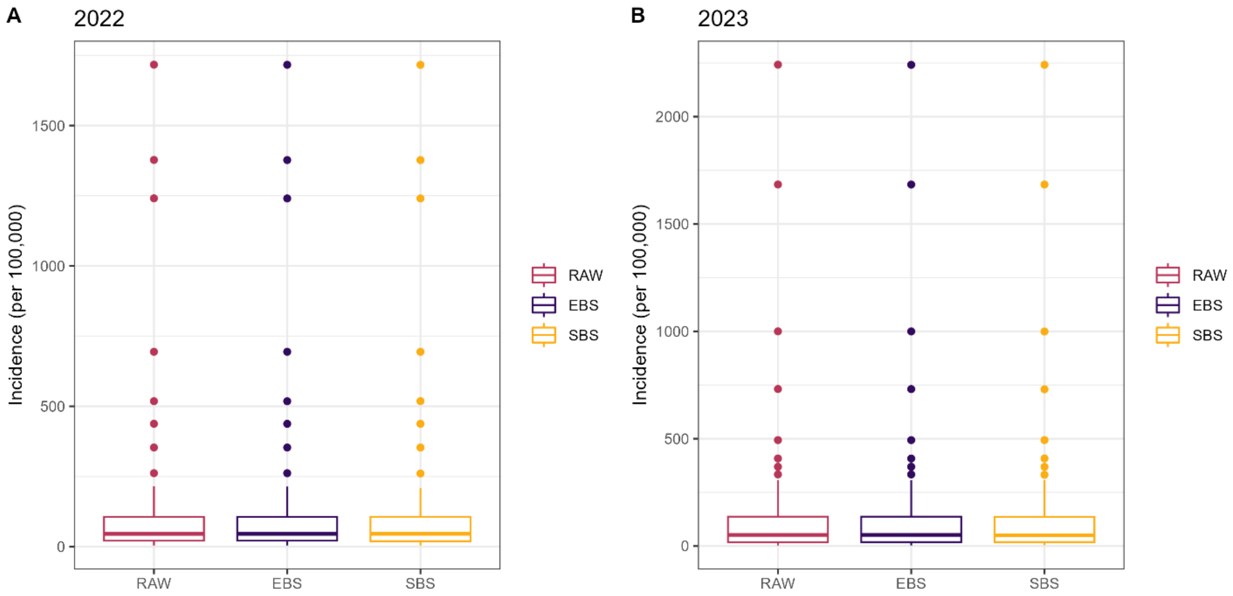

Figure S1: Boxplot comparison of incidence rates for A. 2022, and B. 2023, showing no appreciable differences among Unsmoothed (RAW), Empirical Bayes Smoothing (EBS), and Spatial Bayes Smoothing (SBS)

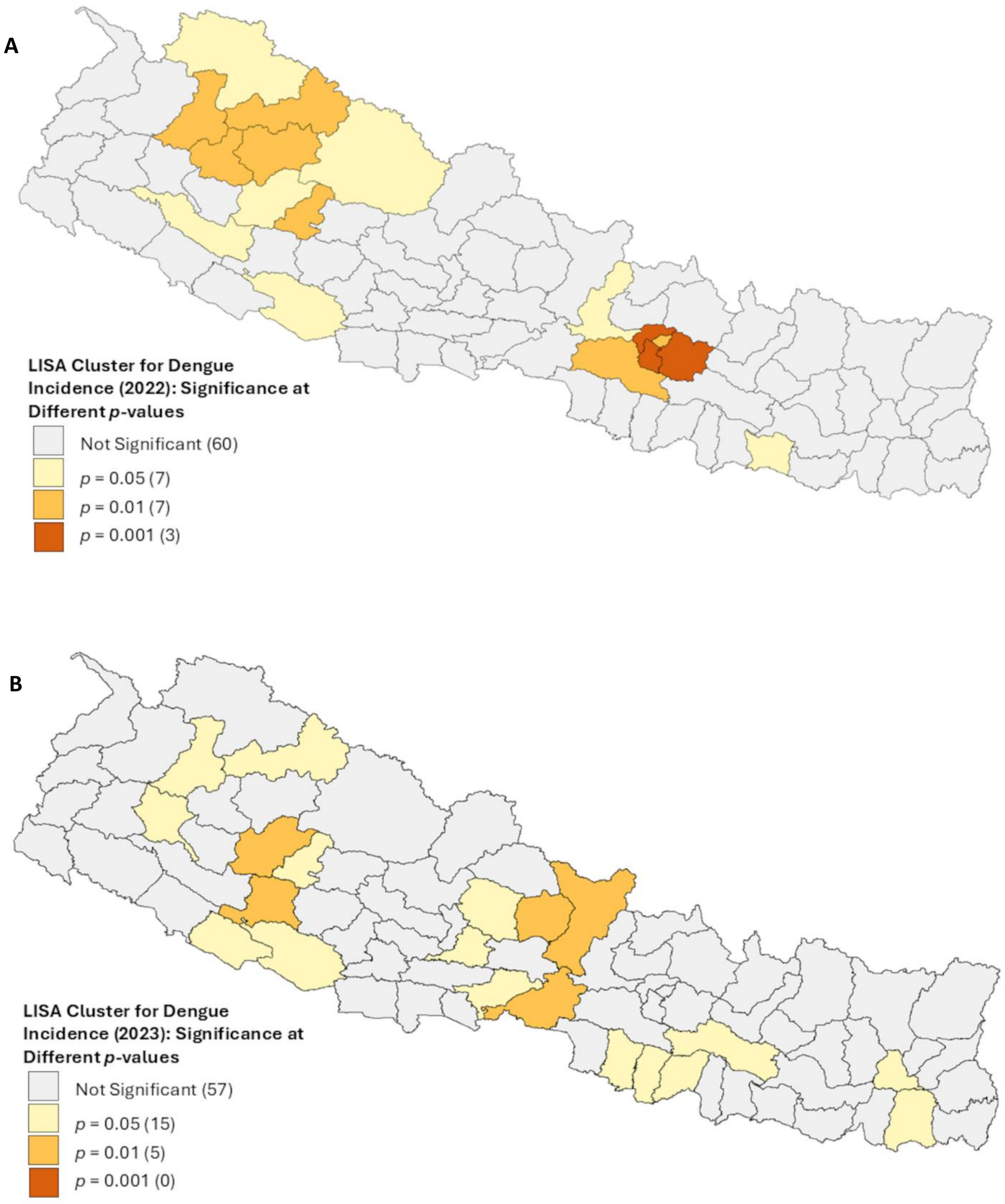

Figure S2: LISA clusters at three significance levels for dengue incidence for A. 2022 and B. 2023
